# Etymologia: *Baylisascaris *

**DOI:** 10.3201/eid1611.ET1611

**Published:** 2010-11

**Authors:** Carol Snarey

**Affiliations:** Author affiliation: Centers for Disease Control and Prevention, Atlanta, GA, USA

**Keywords:** Etymologia, *Baylisascaris*, nematodes, H.A. Baylis

## [ba′′lis-as′kə-ris]

From the Greek term for intestinal worms, *askaris. *This genus of nematodes was named after H.A. Baylis, a parasitologist at the British Museum of Natural History, London, who studied these organisms in the 1920s and 1930s. The most common cause of baylisascariasis in humans and animals is infection with the roundworm *Baylisascaris procyonis, *which takes its name from* Procyon, *a genus of raccoons. The species was first isolated from raccoons in the New York Zoological Park in 1931.

**Source:** Gavin PJ, Kazacos KR, Shulman ST. Baylisascariasis. Clin Microbiol Rev. 2005;18:703–18.

